# K092A and K092B, Two Peptides Isolated from the Dogfish (*Scyliorhinus canicula* L.), with Potential Antineoplastic Activity Against Human Prostate and Breast Cancer Cells

**DOI:** 10.3390/md17120672

**Published:** 2019-11-28

**Authors:** Adrien Bosseboeuf, Amandine Baron, Elise Duval, Aude Gautier, Pascal Sourdaine, Pierrick Auvray

**Affiliations:** 1 Normandy University, University of Caen Normandy (UNICAEN), Sorbonne University, French National Museum of Natural History (MNHN), University of Antilles (UA), French National Centre for Scientific Research (CNRS), French National Institute for Sustainable Development (IRD), Biology of Aquatic Organisms and Ecosystems (BOREA) Research Unit, Sciences Department, CS14032, 14032 CAEN CEDEX 5, France; adrien.bosseboeuf@gmail.com (A.B.); aude.gautier@unicaen.fr (A.G.); 2 Group CELLIS PHARMA, Parc Technopolitain Atalante Saint Malo, 35400 Saint Malo, France; a.baron@kelia-pharma.com (A.B.); e.duval@kelia-pharma.com (E.D.)

**Keywords:** cancer, antineoplastic, ZR-75-1, MDA-Pca-2b, marine peptide, lesser spotted dogfish (*Scyliorhinus canicula*)

## Abstract

Cancer therapy is currently a major challenge within the research community, especially in reducing the side effects of treatments and to develop new specific strategies against cancers that still have a poor prognosis. In this context, alternative strategies using biotechnologies, such as marine peptides, have been developed based on their promise of effectivity associated with a low toxicity for healthy cells. The purpose of the present paper is to investigate the active mechanism of two peptides that were isolated from the epigonal tissue of the lesser spotted dogfish *Scyliorhinus canicula* L., identified NFDTDEQALEDVFSKYG (K092A) and EAPPEAAEEDEW (K092B) on the in vitro growth inhibition of ZR-75-1 mammary carcinoma cells and MDA-Pca-2b prostate cancer cells. The effects of the peptides on cell proliferation and cell death mechanisms were studied by the flow cytometry and immunofluorescence microscopy approaches. The results have shown the onset of both K092A- and K092B-induced early cytoskeleton changes, and then cell cycle perturbations followed by non-apoptotic cell death. Moreover, impedance perturbation and plasma membrane perforation in ZR-75-1 K092A-treated cell cultures and autophagy inhibition in MDA-Pca-2b K092B-treated cells have been observed. In conclusion, these two bioactive peptides from dogfish exhibit antineoplastic activity on the human prostate and breast cancer cells in vitro.

## 1. Introduction

Bioactive peptides are promising for therapeutic use due to several advantages: they are of small size and often constitute the functional part of proteins and, therefore, they are highly functionally efficient, easily synthesized, and modified to enhance their properties; they often specifically target cancer cells without damaging normal cells; they do not accumulate in specific organs; and, their degradation generates amino acids, which minimizes their toxicity and side effects and they are not usually genotoxic [1,2,3]. However, their rapid clearance is a disadvantage for long-term activity [4]. Within the group of bioactive peptides, there is a big diversity of action mode, such as the inhibition of cell migration, inhibition of angiogenesis, oxidative regulation, plasma membrane disruption, cytoskeleton perturbation, inhibition of gene expression, cell-cycle interruption, induction of cell death by apoptosis or necrosis, and modulation of extracellular microenvironment [3,5,6,7]. Several binding peptides can also be used as chemotherapy- or nanoparticles- carriers for targeted drug delivery in cancer therapy due to their strong ability to target and bind specific proteins [8].

**Figure A1:**
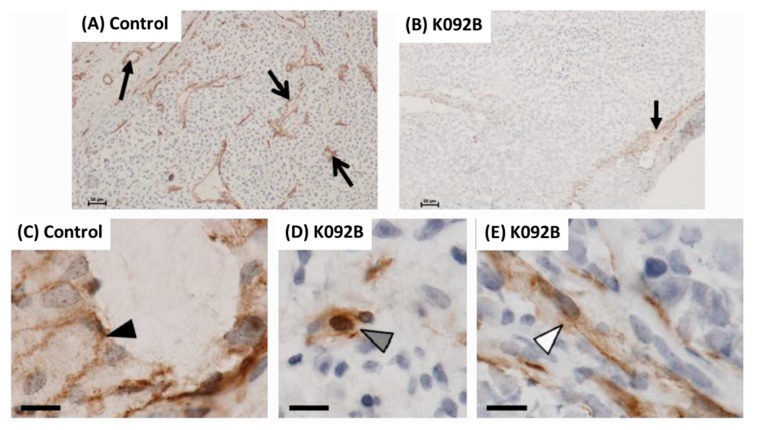
Immunohistochemistry of CD31 in tumors. Expression of CD31 (marker of angiogenesis) on frozen sections of tumors after being implanted into nude mice distributed in two groups: treated with saline buffer PBS (**A**,**C**); treated with K092B (130 mg/kg) (**B**,**D**,**E**). Representative observations of control tumors (**A**) and K092B-treated tumors (**B**) revealing the difference between peripheral expression (full black arrow) and central expression (open black arrows). At higher magnification, representative observations revealing differences between cubic cells (black arrowhead, (**C**)), isolated cells (gray arrowhead, (**D**)) and cells forming a tubular structure (white arrowhead, (**E**)). Scale bars represented 100 µm (**A**–**B**) and 10 µm (**C**–**E**).

There is a recent and strong willing from the scientific community to take advantage from this huge marine biodiversity to improve health science by identifying and characterizing bioactive peptides from marine organisms when considering that 70.8% of the earth surface is covered by aquatic ecosystems. For example, marine organisms are an interesting source of therapeutic peptides, such as peptides with antiseptic, antihypertensive, neuroprotective, cardioprotective, anti-diabetic, analgesic, immunomodulatory, and importantly antitumorale and cytotoxic activities [9,10]. Those peptides are usually derived from different organisms, mainly cyanobacterias, fungi, algae, sponges, tunicates, and mollusks [6,9,11,12]. Interestingly, peptides that were isolated from the deep-sea sponge *Geodia baretti*, illustrated the interest of ecologically and phylogenetic guided search for molecules [13]. Few anti-cancer peptides have been characterized from fishes. In teleosts, Pardaxin has been isolated from the sole *Pardachirus marmoratus* [14], Syngnathusin from the pipefish *Syngnathus acus* [15], Epinecidine-1 from the grouper *Epinephelus coioides* [16], two MCF-7 cells inhibitor peptides from the tuna *Thunnus tonggol* [17], and the YALRAH peptide from the anchovy *Setipinna taty* [18]. Initially isolated from the spiny dogfish *Squalus acanthias*, the aminosterol squalamine exhibiting broad-spectrum antimicrobial activity and blocking angiogenesis that is associated to tumors or to the age-related macular degeneration (AMD), highlights the potential of elasmobranchs’ natural compounds [19,20,21,22]. In this phylum, some bioactive peptides of interest have been identified, as exemplified the sHRSF (shark liver Hepatocyte Regeneration Stimulatory Factor) from *Chiloscyllium plagiosum* [23] and others with angiotensin I-converting enzyme (ACE) inhibitory, antioxidant, antiangiogenic, and anticancer activity [24,25,26,27,28,29]. In animals, anticancer peptides are found in different tissues, including the immune system [30]. Elasmobranchs possess specific lymphomyeloid tissues, including the epigonal tissue associated with the gonads that plays significant roles in immune system development and function, and that is a source of tumor cell inhibitors [31,32]. In a previous report, we have shown that peptides that were isolated from male genital tract of the lesser spotted dogfish *Scyliorhinus canicula* presented a dose-dependent antineoplastic activity on various human cancer cell lines [33]. From those peptides, two have been isolated from epigonal tissue. The first one, K092A, has shown an inhibition of the in vitro growth of MCF-7 (human breast adenocarcinoma; IC50 of 1.09 µg/µL), CCRF CEM (Caucasian acute lymphoblastic leukaemia; IC50 of 0.96 µg/µL), PC3 (Caucasian prostate adenocarcinoma; IC50 of 1.7 µg/µL), and the ZR-75-1 (Human Caucasian breast carcinoma; IC50 of 1.22 µg/µL) cancer cells at 96h post-treatment (WST-1 assay) [31]. The other peptide K092B also presented an inhibition of the in vitro growth of NCI H69 (human carcinoma, small cell lung cancer; IC50 of 1.13 µg/µL), SK-OV-3 (human ovarian carcinoma; IC50 of 1.16 µg/µL), A375 (Human malignant melanoma; IC50 of 1.25 µg/µL), CCRF CEM (IC 50 of 2.2 µg/µL), ZR-75-1 (IC50 of 2.4µg/µL), and MDA-Pca-2b (androgen-independent adenocarcinoma of the prostate; IC50 of 1.3 µg/µL) cancer cells at 96 h post-treatment (WST-1 assay) [33]. In addition, K092A and K092B also showed in the vivo inhibition of cell-derived tumor in Nude mice model without presenting acute toxicity (tested up to 200 and 300 mg/kg for K092A and K092B, respectively) or mutagenic effect (Ames assay) on normal cells [33] (Appendix A, Figure A1).

The purpose of this work was to understand how K092A and K092B are able to inhibit in vitro the growth of ZR-75-1 and MDA-PCa-2b cell lines, respectively. We first realized a kinetic study from 6 h to 96 h post-treatment to evidence the first noticeable effects. We then studied cell proliferation and cell death mechanisms by flow cytometry and cytoskeleton integrity, and the cell characteristics by immunofluorescence. Our results have shown that K092A induced drastic electric impedance variation in cultures, early cytoskeleton perturbation, inhibition of cell proliferation, membrane destabilization, and necrosis. K092B induced cytostatic effect, autophagy inhibition, cytoskeleton perturbation, and non-apoptotic cell death. Interestingly, the action mode of both peptides starts with the induction of cytoskeleton disruption. This event seems to drive the growth inhibition for ZR-75-1 and MDA-Pca-2b cells through different ways. Finally, this work confirms that marine organisms are a good source of bioactive peptides and emphasizes the fact that dogfish is a potent source of antineoplastic peptides.

## 2. Results

### 2.1. Decrease in Mitochondrial Activity and Cell Number Was Reported in K092A-Treated Human Mammary Carcinoma and K092B-Treated Human Prostate Cancer Cells

**Figure 1:**
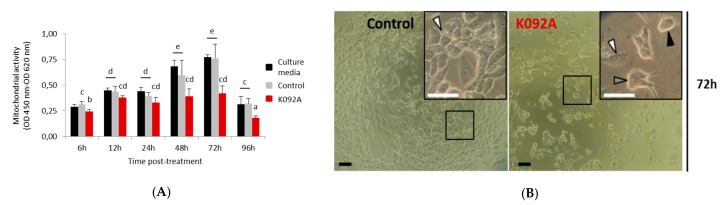
ZR-75-1 cells treated with K092A. (**A**) Mitochondrial activity measured by using the WST-1 colorimetric assay (OD 450 nm–OD 620 nm) at 6 h, 12 h, 24 h, 48 h, 72 h and 96 hpt under three different conditions of cell culture: culture media (black bars), culture media with 0.01 M ammonium bicarbonate (control, grey bars), culture media with K092A at 1.22 µg/µL (red bars). Data were obtained from three independent experiments (*N* = 3; *n* = 6) and statistical analyses were performed using the Mann and Whitney test (*p* < 0.05 between each a to e statistical groups). (**B**) Representative light microscopic observations of ZR-75-1 control cells and K092A-treated cells at 72 hpt. Inserts were focused so as to distinguish adherent cells (white arrows) from cells presenting an abnormal morphology (grey arrow) and round suspended cells (black arrow). Both bars represented 40 µm.

**Figure 2:**
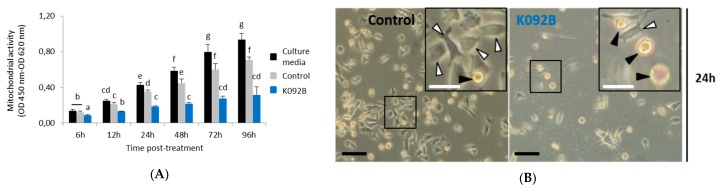
MDA-Pca-2b cells treated with K092B. (**A**) Mitochondrial activity measured by using the WST-1 colorimetric assay (OD 450 nm–OD 620 nm) at 6 h, 12 h, 24 h, 48 h, 72 h and 96 hpt under three different conditions of cell culture: culture media (black bars), culture media with 0.01 M ammonium bicarbonate (control, grey bars), culture media with K092B at 1.3 µg/µL (blue bars) in 0.01 M ammonium bicarbonate. Data was obtained from three independent experiments (*N* = 3; *n* = 6) and statistical analyses were performed using the Mann and Whitney test (*p* < 0.05 between each a to g statistical groups). (**B**) Representative light microscopic observations of MDA-Pca-2b control cells and K092B-treated cells at 24 hpt. Inserts were focused so as to distinguish adherent cells (white arrows) from round suspended cells (black arrows). Both bars represented 40 µm.

The mitochondrial activity of the cell culture was measured while using the WST-1 test at 6 h, 12 h, 24 h, 48 h, 72 h, and 96 hours post-treatment (hpt) on ZR-75-1 (Figure 1) and MDA-Pca-2b (Figure 2) cells grown with: (i) culture media, (ii) culture media and 0.01 M ammonium bicarbonate, and (iii) culture media and K092A (Figure 1A) or K092B (Figure 2B) dissolved in 0.01 M ammonium bicarbonate at the final concentration that corresponded to the IC50. This assay showed a gradual increase of the mitochondrial activity in both controls and for the two types of cells, reflecting their proliferation over time with one exception for the ZR-75-1 cells at 96 hpt. A significant decrease of the mitochondrial activity for K092A-treated ZR-75-1 cells when compared to the ammonium bicarbonate control was observed at 6 hpt (0.245 ± 0.017 for treated vs. 0.312 ± 0.029 for control), from 48 hpt (0.394 ± 0.050 for treated vs. 0.597 ± 0.145 for control) and until 96 hpt (0.183 ± 0.021 for treated vs. 0.321 ± 0.047 for control) (Figure 1). Significantly, a decrease by half of the mitochondrial activity for K092B-treated MDA-Pca-2b cells when compared to the ammonium bicarbonate control was observed each time, from 6 hpt (0.088 ± 0.005 for treated vs. 0.137 ± 0.014 for control) and until 96 hpt (0.316 ± 0.088 for treated vs. 0.708 ± 0.067 for control) (Figure 2A). Furthermore, microscopic observations at 6 h, 48 h, 72 h, and 96 hpt revealed that the K092A-treated ZR-75-1 cells exhibited a decrease in the number of cells as well as the presence of more round suspended cells and abnormal cells, as illustrated at 72 hpt (Figure 1B). Microscopic observations also showed that the K092B-treated MDA-Pca-2b cells presented a decrease of the cell number as well as the presence of more round suspended cells, as illustrated at 24 hpt (Figure 2B). 

These results illustrate the potential in vitro antineoplastic activity of K092A and K092B on the ZR-75-1 cells and MDA-Pca-2b cells, respectively.

### 2.2. K092A- and K092B-Induced Perturbation of Electric Impedance in Culture Plates of Treated Cells

**Figure 3:**
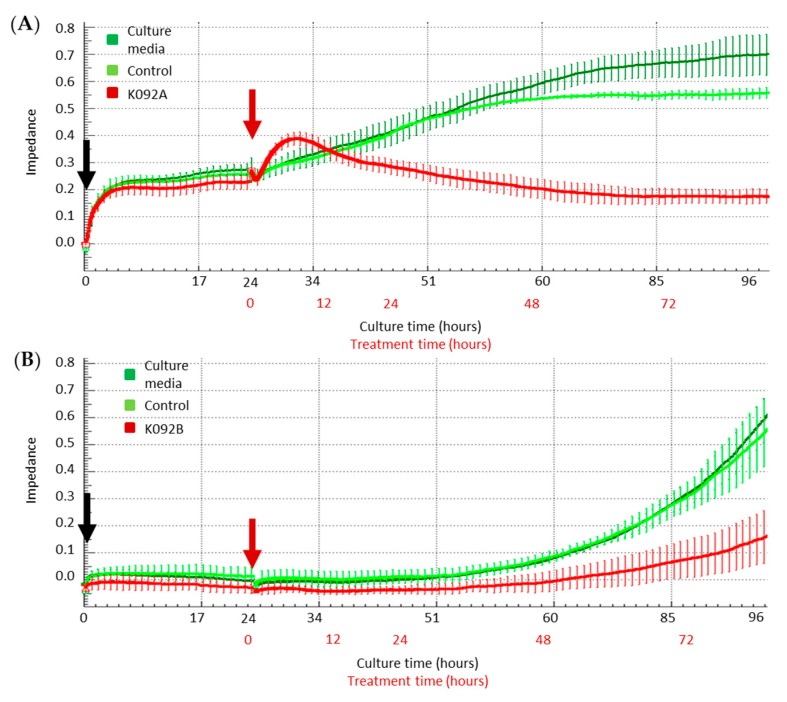
Real-time electric impedance in culture plates of cells treated with K092A or K092B. Real time analysis of impedance in cultures of control cells (dark and light green curves) and of K092A-treated ZR-75-1 cells (**A**) (red curve) or K092B-treated MDA-Pca-2b cells (**B**) (red curve) during the first 96 h of culture time (black numbers) and during 72 h in presence of treatment (red numbers). Cells were seeded (black arrow) 24 h before the treatment (red arrow) and results were represented by mean ± SD of 4 replicates.

Real-time electric impedance sensing measurements of culture plates were investigated from the time of cell seeding until 72 hpt while using the XCelligence system for K092A-treated ZR-75-1 cells (Figure 3A) or for K092B-treated MDA-Pca-2b cells (Figure 3B). All culture conditions presented the same impedance until the treatment time and both of the controls exhibited a similar increase of impedance, even if the ammonium bicarbonate control reached an amplitude that was slightly lower than the one observed for the culture media control from 45 h until 72 hpt (Figure 3A). When compared with the controls, K092A-treated cells presented a fast increase of impedance from treatment time until 6 hpt, followed by a decrease until 48 hpt. Subsequently, impedance was stabilized at the corresponding initial level until the end of the culture time (Figure 3A). For K092B-treated MDA-Pca-2b cells culture, the profile of impedance was similar to the ones that were observed for controls, but of lower amplitude (Figure 3B). 

These data show that K092A and K092B have induced a perturbation of the electrical impedance in the ZR-75-1 and MDA-Pca-2b cell cultures, respectively. The different profiles observed suggested that the two peptides drive the perturbation of cell adhesion through different ways.

### 2.3. K092A and K092B Induced Perturbation of the Cell Cycle Repartition

**Figure 4:**
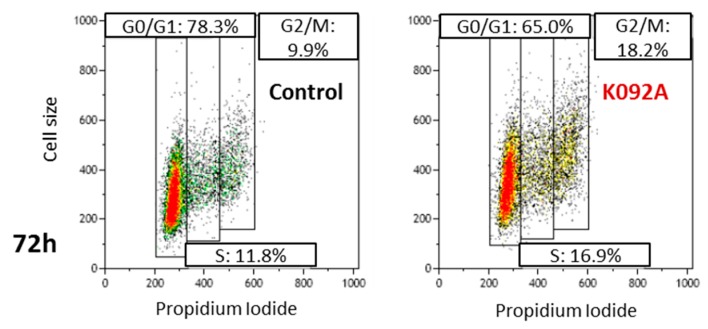
Representative flow cytometry data at 72 hpt for the control and K092A-treated cells.

**Figure 5:**
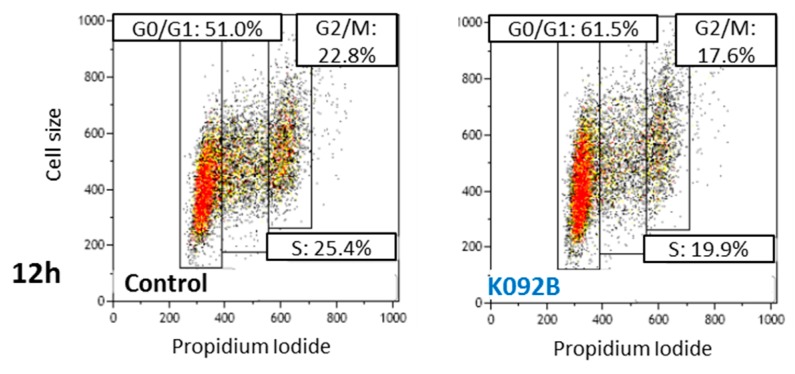
Representative flow cytometry data at 12 hpt for control and K092B-treated cells.

Cell cycle repartition was investigated by flow cytometry at 4 h, 8 h, 12 h, 24 h, 48 h, and 72 hpt on K092A-treated ZR-75-1 cells (Table 1, Figure 4) and K092B-treated MDA-Pca-2b cells (Table 2, Figure 5) and then compared to the controls. At 4 hpt, no significant difference was reported for K092A- or K092B-treated cells and at 8 hpt and 12 hpt for K092A-treated cells (Table 1 and Table 2, Figure 4 and Figure 5). At 24 hpt, less K092A-treated ZR-75-1 cells were in the S phase (11.5% ± 1.9% for treated vs. 15.5% ± 1.5% for control). At 48 hpt, more cells of the population were in G0/G1 (80.2% ± 1.0% for treated vs. 74.3% ± 1.0% for control) and fewer cells in S (8.9% ± 0.9% for treated vs. 14.0% ± 0.8% for control) and G2/M (8.7% ± 0.8% for treated vs. 11.8% ± 1.2% for control), (Table 1). At 72 hpt, the opposite was observed with fewer K092A-treated ZR-75-1 cells in G0/G1 (66.6% ± 2.2% for treated vs. 78.0% ± 1.2% for control) and more cells in S (14.9% ± 2.7% for treated vs. 8.7% ± 2.4% for control) and G2/M (16.6% ± 2.5% for treated vs. 9.0% ± 1.8% for control) (Table 1, Figure 4). 

The cell cycle repartition of the K092B-treated MDA-Pca-2b cell population presented early perturbation, with more cells in G0/G1 at 8 hpt (64.5% ± 0.6% for treated vs. 59.2% ± 1.3% for control) and 12 hpt (60.0% ± 2.1% for treated vs. 49.8% ± 1.8% for control), fewer cells in S at 8 hpt (14.3% ± 1.3% for treated vs. 22.4% ± 0.4% for control), and at 12 hpt (17.4% ± 2.2% for treated vs. 22.9% ± 2.5% for control) and fewer cells in G2/M at 12 hpt (19.0% ± 1.9% for treated vs. 26.7% ± 3.7% for control) (Table 2, Figure 5). At 24 hpt, the K092B-treated MDA-Pca-2b cell population comprised fewer cells in G0/G1 (60.5% ± 0.3% for treated vs. 66.8% ± 0.6% for control), as well as at 48 hpt (69.7% ± 1.1% for treated vs. 72.5% ± 0.7% for control) and more cells in G2/M (21.5% ± 0.7% for treated vs. 16.8% ± 0.3% for control) Finally, at 72 hpt, the K092B-treated cells comprised fewer cells in S (10.2% ± 1.1% for treated vs. 15.2% ± 0.3% for control) (Table 2).

These results show that K092A and K092B have promoted an antiproliferative effect, starting at 24 hpt in ZR-75-1 cells and at 8 hpt in MDA-PCa-2b cells, respectively.

### 2.4. K092A Induced Cytoskeleton Perturbation Followed by Membrane Destabilization and Necrosis in ZR-75-1 Cells

**Figure 6:**
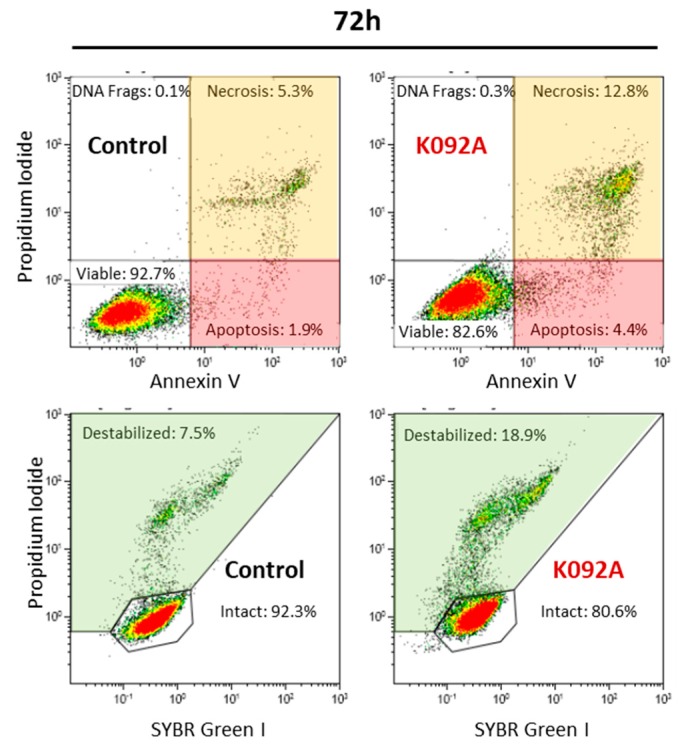
Representative flow cytometry data set at 72 hpt for ZR-75-1 control and K092A-treated cells, annexin V/propidium iodide staining for apoptosis and necrosis, SYBR Green I/propidium iodide staining for membrane integrity study. Significant results were highlighted for apoptosis (light red box), necrosis (light orange box), and destabilized membranes (light green box).

**Figure 7:**
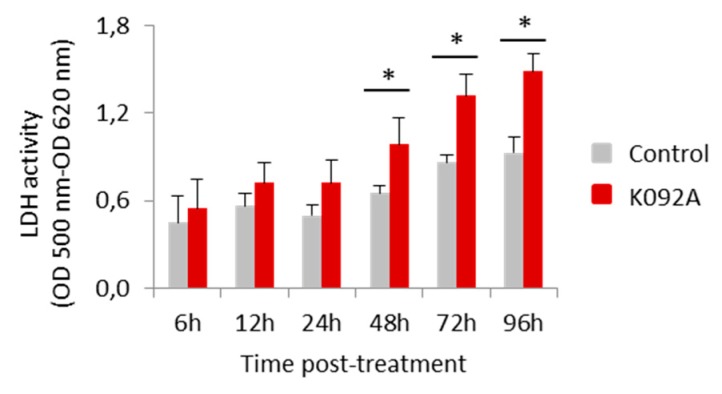
Activity of the lactate dehydrogenase released in the extracellular medium by ZR-75-1 cells. Activity was measured using the LDH assay (OD 500 nm–OD 620 nm) at 6 h, 12 h, 24 h, 48 h, 72 h, and 96 hpt for the control cells (greys bars) and K092A-treated cells (red bars). Data was obtained from three independent experiments (*N* = 3; *n* = 6), represented by mean ± SD and statistical analysis was performed while using the Mann and Whitney test (* *p* < 0.05, treated vs. untreated control).

**Figure 8:**
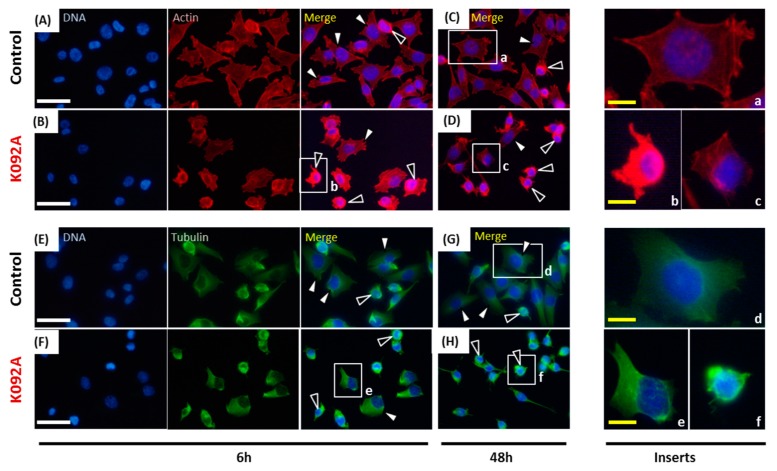
Actin and tubulin aggregations in K092A-treated ZR-75-1 cells. (**A**–**D**) Actin was stained using phalloidin and nuclei using DAPI at 6 hpt (**A**,**B**) and 48 hpt (**C**,**D**) for control cells (**A**,**C**) and K092A-treated cells (**B**,**D**). Inserts (a–c) were focused in order to distinguish spread and adherent normal cells (white arrows) from cells with agglutinated actin (black arrows). (**E**–**H**) Tubulin was stained using anti-tubulin antibody and nuclei using DAPI at 6 hpt (**E**,**F**) and 48 hpt (**G**,**H**) for control cells (**E**,**G**) and K092A-treated cells (**F**,**H**). Inserts (d–f) were focused in order to distinguish spread and adherent normal cells (white arrows) from cells with agglutinated tubulin (black arrows). The white scale bar is 40 µm and yellow bar 10 µm.

Cell death mechanisms (Table 3, Figure 6 and Figure 7) and cytoskeleton integrity (Figure 8) were investigated at 24 hpt, 48 hpt and 72 hpt and at 6 hpt and 48 hpt, respectively, to further explore the mechanism of K092A involved in impedance perturbation and antiproliferative effect.

K092A-treated ZR-75-1 cell population: the level of destabilized membranes increased 1.5 fold at 48 hpt (8.4% ± 1.1% for treated vs. 5.4% ± 1.5% for control), three-fold at 72 hpt (17.6% ± 3.3% for treated vs. 6.5% ± 1.3% for control), and a three-fold increase in necrotic cells was evidenced at 72 hpt (10.8% ± 1.9% for treated vs. 3.3% ± 1.0% for control) (Table 3, Figure 6). The activity of LDH released in extracellular medium was assessed at 6 h, 12 h, 24 h, 48 h, 72 h, and 96 hpt in order to confirm this membrane destabilization process observed by using flow cytometry. K092A induced a 1.5 fold increase of LDH activity at 48 h (0.987 ± 0.184 for treated vs. 0.655 ± 0.049 for control), 72 h (1.318 ± 0.147 for treated vs. 0.864 ± 0.053 for control), and at 96 hpt (1.484 ± 0.120 for treated vs. 0.928 ± 0.107 for control) (Figure 7). 

When the cytoskeleton of cells was examined, the population that was treated with K092A presented more cells with the aggregation of actin and fewer adherent cells at 6 hpt (Figure 8A,B) and at 48 hpt (Figure 8C,D). Similar observations were made for tubulin at 6 hpt (Figure 8E,F) and at 48 hpt (Figure 8G,H). 

These results show that K092A has driven a drastic cytoskeleton perturbation, followed by membrane destabilization and necrosis. 

### 2.5. K092B Induced Prolonged Necrosis and Membrane Destabilization in MDA-Pca-2b Cells 

**Figure 9:**
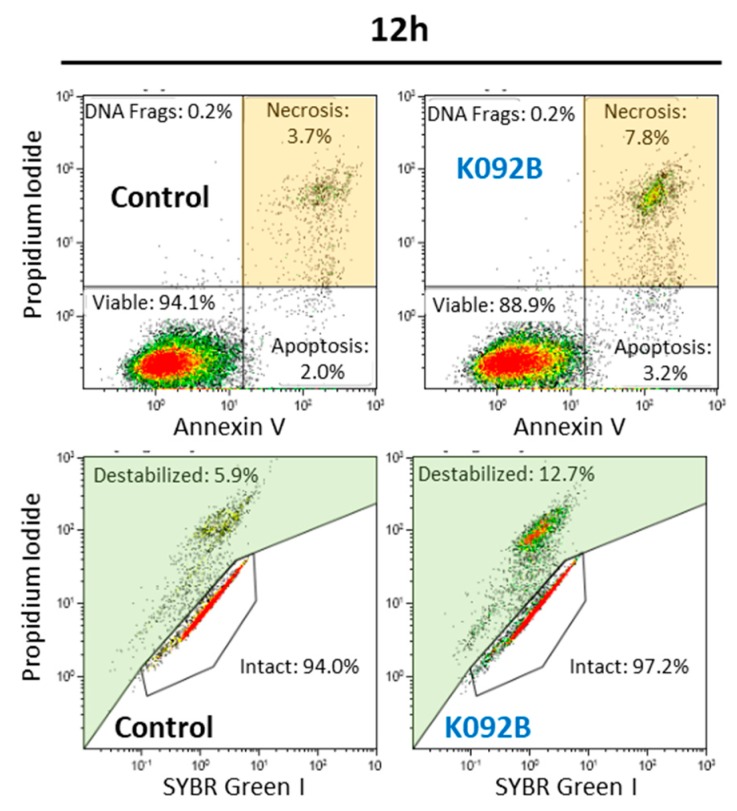
Representative flow cytometry data set at 12 hpt for MDA-Pca-2b control and K092B-treated cells, annexin V/propidium iodide staining for apoptosis and necrosis, SYBR Green/propidium iodide staining for membrane integrity. Significant results were highlighted for necrosis (light orange box) and destabilized membranes (light green box).

Apoptosis, necrosis, and membrane destabilization were investigated at 4 h, 8 h, 12 h, 24 h, 48 h, and 72 hpt in order to correlate the antiproliferative effect and the cell death mechanisms induced by K092B on MDA-Pca-2b cells (Table 4, Figure 9).

The K092B-treated MDA-Pca-2b cell population presented twice as many necrotic cells at 8 hpt (4.6% ± 0.5% for treated vs. 2.8% ± 1.1% for control), three times more necrotic cells at 12 hpt (12.7% ± 4.1% for treated vs. 3.9% ± 0.9% for control), and a two-fold increase in the number of necrotic cells at 72hpt (6.5% ± 0.8% for treated vs. 3.5% ± 1.7% for control) when compared to the control cells (Table 4, Figure 9). Membrane destabilization also increased 1.5 fold in K092B-treated cells at 12 hpt (11.5% ± 1.8% for treated vs. 7.0% ± 0.9% for control), and two-fold at 72 hpt (7.6% ± 0.3% for treated vs. 3.7% ± 0.2% for control). More cell fragments were also observed in the K092B-treated cells at 48 hpt (5.7% ± 0.7% for treated vs. 2.9% ± 0.1% for control) and at 72 hpt (5.9% ± 0.7% for treated vs. 3.4% ± 0.1% for control) (Table 4, Figure 9).

These results show that K092B has induced cell death by membrane destabilization and necrosis. 

### 2.6. K092B Induced Early Autophagy Inhibition, Increase of Neutral Red Retention and Cytoskeleton Perturbation in MDA-Pca-2b Cells 

**Figure 10:**
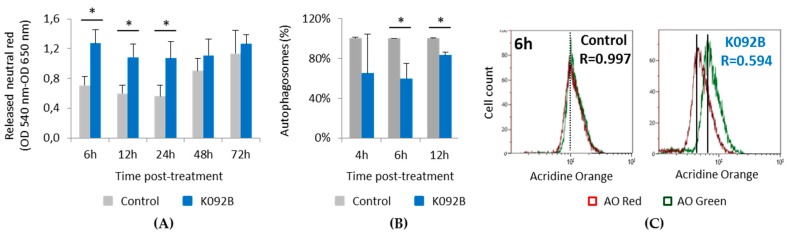
Neutral red retention and autophagy inhibition in K092B-treated MDA-Pca-2b cells. (**A**) Released neutral red was measured by the neutral red assay (DO 570 nm–DO 620 nm) in control cells (grey bars) and K092B-treated cells (blue bars) at 6 h, 12 h, 24 h, 48 h, and 72 hpt. Data was obtained from three independent experiments (*N* = 3; *n* = 6), represented by mean ± SD and statistical analyses were performed using Mann and Whitney test (* *p* < 0.05, treated vs. untreated control). (**B**) Percentages of autophagosomes in control cells (grey bars) and K092B-treated cells (blue bars) measured by flow cytometry using acridine orange assay (AO) at 4 h, 6 h, and 12 hpt. Statistical analysis was performed while using the Mann and Whitney test (* *p* < 0.01, treated vs. untreated control). Data was obtained from two independent experiments. (**C**) Representative flow cytometry data set at 6 h post-treatment and corresponding *R* value of autophagosomes percentages (*R* = red fluorescence due to AO binding to autophagosomes/green fluorescence due to AO binding to nucleic acids).

**Figure 11:**
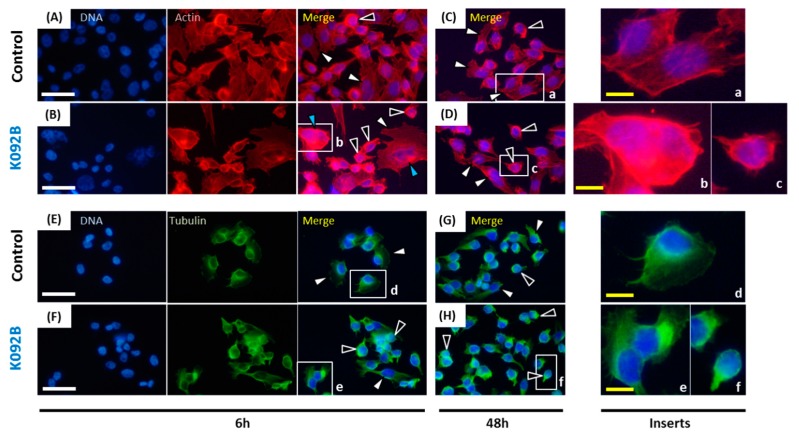
Actin and tubulin aggregations in K092B-treated MDA-Pca-2b cells. (**A**–**D**) Actin was stained using phalloidin and nuclei using DAPI at 6 hpt (**A**,**B**) and 48 hpt (**C**,**D**) for control cells (**A**,**C**) and K092B-treated cells (**B**,**D**). Inserts (a–c) were focused in order to distinguish spread and adherent normal cells (white arrows) from cells with agglutinated actin (black arrows) and multinucleated cells (blue arrows). (**E**–**H**) Tubulin was stained using anti-tubulin antibody and nuclei using DAPI at 6 hpt (**E**,**F**) and 48 hpt (**G**,**H**) for the control cells (**E**,**G**) and K092B-treated cells (**F**,**H**). Inserts (d–f) were focused in order to distinguish spread and adherent normal cells (white arrows) from cells with agglutinated tubulin (black arrows). The white scale bar is 40 µm and yellow bar 10 µm.

The neutral red retention capacity of lysosomes was studied at 6 h, 12 h, 24 h, 48 h, and 72 hpt (Figure 10A), autophagy at 4 h, 6 h, and 12 hpt (Figure 10B,C), as well as cytoskeleton integrity at 6 h and 48 hpt in order to further explore the mechanism involved in early antiproliferative and cell death effect observed in K092B-treated cells (Figure 11).

In the control cells, the increase of neutral red retention capacity that was observed between 24 hpt and 72 hpt seems to correlate with the increase of cell number during the same period. K092B-treated cells presented a two-fold increase in the lysosome retention capacity at 6 hpt (1.273 ± 0.181 for treated vs. 0.707 ± 0.123 for control), 12 hpt (1.084 ± 0.179 for treated vs. 0.599 ± 0.106 for control), and at 24 hpt (1.069 ± 0.230 for treated vs. 0.560 ± 0.153 for control) when compared to the control cells (Figure 10A). Autophagy study also showed that K092B-treated cells presented fewer autophagosomes than control cells at 6 hpt (60% ± 15% for treated vs. 100% for control) and 12 hpt (83% ± 3% for treated vs. 100% for control) (Figure 10B,C). 

When the cytoskeleton of cells was examined, the population that was treated with K092B presented more cells with aggregated actin cytoskeleton and fewer adherent cells at 6 hpt (Figure 11A,B) and at 48 hpt (Figure 11C,D). Similar observations were made for tubulin cytoskeleton at 6 hpt (Figure 11E,F) and 48 hpt (Figure 11G,H). Moreover, the presence of multinucleated cells was also detected in K092B-treated cells at 6 hpt (Figure 11B).

These results show that K092B induced early cytoskeleton perturbation and autophagy inhibition.

## 3. Discussion

This study reported that the first effects that were observed for K092A treatment in ZR-75-1 cells were a durable perturbation in electric impedance of cell culture and a perturbation of the cytoskeleton. These events are quickly followed by a slowing-down of the cell cycle, an important cell membrane destabilization, and finally, cell death by necrosis. The X-Celligence system has been previously used to study the kinetics and effects of peptides or drugs on cells, since electric impedance was correlated with various cell properties, such as cell size, cell proliferation rate, cell adherence intensity, and cell number [34,35,36,37,38]. In the present study, the rapid and transient increase of cell impedance during the first 24 h of treatment of ZR-75-1 cells suggested quick cytoskeletal changes, as observed by phenethyl isothiocyanate [38] or nocodazole in lung cancer cells [36]. Moreover, the late stability of impedance suggested a durable antiproliferative effect. These results appear to be correlated with cytoskeleton perturbation, as observed for both actin and tubulin cytoskeletons at 6 hpt and 48 hpt, and allow for us to exclude an increase of cell junction formation, which could also be responsible for the transient increase of cell impedance, because of the decrease of cell size and cell interactions observed. Such antiproliferative effects resulting on cytoskeleton disruption have been described after microtubule polymerization disruption induced by cucurbitacin B in human breast cancer cells [39] or after actin filaments reorganization induced by ophiobolin A in human glioblastoma cells [40]. In the present study, an antiproliferative effect has also been reported by WST-1 assay and flow cytometry analyses, which indicate that cell proliferation strongly decreased in K092A-treated ZR-75-1 cells as soon as 48 hpt. When considering the close relationship between cell cycle completion and cytoskeleton dynamics and the timing of observed antiproliferative effects and cytoskeleton perturbation, we could hypothesize that K092A affects the cell cycle progression of ZR-75-1 cells through the alteration of their cytoskeleton network. Indeed, direct perturbation of the dynamics of microtubule induced cell cycle arrest before metaphase often associated with mitotic spindle aberrations, as reported in many studies on various cancer cell lines [39,41,42]. Finally, the membrane perforation that was observed in K092A-treated cells seems to trigger necrotic death due to the interactions between cytoskeleton and plasma membrane [43] and could result from cytoskeleton disruption. 

This study also reported that the first noticeable effects of K092B on MDA-Pca-2b cells, as observed at 6 hpt, consisted of: a cytostatic effect that was observed by real-time electric impedance sensing measurements, autophagy inhibition, and cytoskeleton aggregation. They were followed by necrosis, membrane destabilization, and the inhibition of cell proliferation. Autophagy is a complex mechanism that allows for the turnover of organelles and of proteins, therefore protecting cells from various stress conditions [44]. In cancer cells, according to its intensity, autophagy can promote either cell survival allowing tumor survival and development, or cell death by apoptosis or necrosis [44,45,46,47,48,49]. The inhibition of autophagy was correlated with an increase of caspase-dependent apoptosis in various cells, such as HeLa or lymphoma cells [48] and necrosis in human glioblastomes [49]. Consistent with these cell behaviors, our results have shown that necrosis features at 8 h and 12 hpt followed the inhibition of autophagy, as observed at 6 hpt, which suggests that K092B induces early MDA-Pca-2b cell death by autophagy inhibition. In light of the various interactions described between cytoskeleton and autophagy [50,51,52], in particular for lysosomes and endosomes trafficking [53], we could hypothesize that the autophagy inhibition that was observed on K092B-treated cells was induced by the cytoskeleton perturbations that were observed at the same time. Such correlation between microtubule remodeling and autophagy inhibition has been previously reported on mouse neural cells [54]. Moreover, the early increase of lysosomal retention capacity that was detected in K092B-treated cells could be correlated to autophagy inhibition and similarly with the cytoskeleton perturbation. Indeed, the fusion of lysosomes with others organelles is needed to form autophagosomes and autolysosomes [46], which suggests that autophagy inhibition might involve an increase in the number of lysosomes due to a lack of fusion. 

Such a mechanism of action, causing cytoskeleton destabilization, cell cycle perturbation, and finally, cell death, has been previously described. For example, the 17-amino acids peptide C36L1 inhibits in vitro the migration, the invasion, and proliferation of melanoma cells by causing direct depolymerization of microtubules [55]. In this study, Figueiredo et al. were able to identify that C36L1 directly binds to the tubulin protein, causing depolymerization [55]. This direct interaction between bioactive peptide and tubulin can also lead to polymerization inhibition, cell cycle arrest, and cell death, as reported for the peptide MMAE [10]. These kinds of peptide are very promising for improved therapy, since, for example, the peptide MMAE has been approved by the U.S. Food and Drugs Administration in 2011 for the treatment of Hodgkin and systemic anaplastic large cell lymphoma [10].

## 4. Materials and Methods 

### 4.1. Cell Culture and Treatments Conditions

The ZR-75-1 cell line was established from the metastatic site of breast glands of a 63 years old caucasian woman with a ductal breast carcinoma (cell line obtained from the global bioresource center ATCC; www.atcc.org/). This cell line was grown in vitro and in vivo and it expressed wild type and a variant of estrogen receptor as well as the progesterone receptor. MDA-PCa 2b-cell line was established from the bone metastasis of a 63 years old black man with androgen-independent adenocarcinoma of the prostate (bioresource center ATCC; www.atcc.org/). The ZR-75-1 cells were maintained in RPMI 1640 media (supplemented with 10% SVF, 2 mM L-Glu, 10 mM beta estradiol, 1 mM Na pyruvate) at 27 °C in a 5% CO_2_ air incubator. MDA-PCa-2b cells were maintained in Ham’s F12 media (supplemented with 20% SVF, 5 mM L-Glu, 10 ng/mL EGF, hydrocortisone 100 pg/mL) at 37 °C in a 5% CO_2_ air incubator. Prior to the experiment, the cells were grown to approximately 80% confluence three times and then exposed to K092A (1.22 µg/µL) or K092B (1.3 µg/µL). K092A (94% purity) and K092B (98% purity) was purchased from the Bionexus company (Strasbourg, France), stored at −20 °C, and then dissolved in bicarbonate ammonium (0.1 M) when needed. The results were compared with those that were obtained for both controls, cells grown in culture media, and in culture media with bicarbonate ammonium (0.1 M). 

### 4.2. Mitochondrial Activity Assay

Mitochondrial activity was measured by the WST-1 colorimetric assay (Roche; Ref.: 11 644 807 001) that was based on the cellular capacity to metabolize the WST-1 by mitochondrial enzymatic complex. The cells were seeded into 96-well plates (ZR-75-1: 7500 cells/well; MDA-Pca-2b: 5000 cells/well) and treated in replicates for 6 h, 12 h, 24 h, 48 h, 72 h, and 96 h. WST-1 was added, as recommended by suppliers, and the plates were incubated for 2 h before being analyzed in a spectrophotometer (BioRad; iMark; Marnes-la-Coquette, France). Optical density (OD) at 620 nm that corresponded to background level was subtracted from OD at 450 nm, corresponding to WST-1 degradation. For each study, statistical analysis was performed while using the Mann and Whitney test (*p* < 0.05) on three independent experiments (*N* = 3; *n* = 9).

### 4.3. Lactate Dehydrogenase Activity Assay

Lactate dehydrogenase (LDH) activity was measured by the LDH cytotoxic colorimetric assay (Roche; Ref.: 11 644 793 001) based on the reduction of NAD by the cytoplasmic LDH that was released in extracellular medium after membranes destabilization. The resulting NADH is utilized in the stoichiometric conversion of a tetrazolium dye into red formazan salt by a catalyst complex that was included in the LDH assay kit. The cells were seeded into 96-well plates (ZR-75-1: 7500 cells/well) and treated in replicates for 6 h, 12 h, 24 h, 48 h, 72 h, and 96 h. The catalyst complex solution was added, as recommended by the suppliers, and the plates were incubated for 30 min. before being analyzed in a spectrophotometer (BioRad; iMark; Marnes-la-Coquette, France). Optical density (OD) at 620 nm, which corresponded to background level, was subtracted from OD at 500 nm, which corresponded to formazan salt detection. Statistical analysis was performed while using the Mann and Whitney test (*p* < 0.01) on three independent experiments (*N* = 3; *n* = 9).

### 4.4. Neutral Red Retention Assay

Neutral red retention capacity was based on the cellular capacity to sequestrate the neutral red inside lysosomes. The cells were seeded into 96-well plates (MDA-Pca-2b: 5000 cells/well) and treated in replicates for 6 h, 12 h, 24 h, 48 h, 72 h, and 96 h. Neutral red was added (0.033%) and incubated with cells for two hours at 37 °C. The cells were washed, fixed (0.5% formaldehyde in PBS), and the neutral red was released from lysosomes while using solubilization solution (1% acetic acid, 50% ethanol in PBS), which was analyzed in a spectrophotometer (BioRad; iMark; Marnes-la-Coquette, France. Optical density (OD) at 650 nm, corresponding to background level, was subtracted from OD at 540 nm corresponding to neutral red. Statistical analysis was performed while using the Mann and Whitney test (*p* < 0.01) on three independent experiments (*N* = 3; *n* = 9).

### 4.5. Apoptosis/Necrosis and Membranes Integrity Analysis

Both apoptosis/necrosis and membrane integrity were quantified by flow cytometry using two double stainings: propidium iodide (PI) and annexin V (AV), or PI and SYBR Green I (SGI) respectively. PI and SGI were both used to stain DNA, but only the SGI could pass through the cellular membranes without perforation. Annexin V allowed for apoptosis/necrosis detection by its high affinity to bind phosphatidylserines that were exposed at the extracellular side of apoptotic and necrotic cells. The cells were seeded into 24 well-plates (ZR-75-1: 44,531 cells/well; MDA-Pca-2b: 29,688 cells/well) and treated in replicates for 4 h, 8 h, 12 h, 24 h, 48 h, and 72 h. The cells were then collected (170 U trypsin/mL), washed, and then stained in two ways: (i) apoptosis/necrosis analysis using PI and AV (both 1:500, as described by suppliers); (ii) membrane integrity analysis while using SGI at 1:1 × 10^6^ and PI at 1:500 with an incubation time of 30 min. in dark conditions. The samples were then analyzed by flow cytometry (Gallios cytometer, Beckman Coulter Life Sciences, Villepinte, France). For each sample, 20,000 events were counted and statistical analyses were performed on three independent experiments while using the Mann and Whitney test (*p* < 0.05) (*N* = 3; *n* = 6). 

### 4.6. Cell Cycle Analysis

Cell cycle repartition was investigated at 4 h, 8 h, 12 h, 24 h, 48 h, and 72 h by flow cytometry with PI staining. On ethanol fixed cells, PI staining allowed for DNA quantification corresponding to cell populations in G0/G1 (2C); S (between 2C and 4C) or G2/M (4C). The cells were seeded in replicates into 24-well plates (ZR-75-1: 44,531 cells/well; MDA-Pca-2b: 29,688 cells/well) and treated. Afterwards, they were collected (170 U trypsin/mL), washed, fixed, and stored in 70% ethanol at −20 °C. Before flow cytometry analysis, the cells were washed at 37 °C and stained for 30 min in dark conditions with PI, as described by suppliers. The samples were then analyzed by flow cytometry (Gallios cytometer with Kaluza licence, Beckman Coulter Life Sciences, Villepinte, France). For each sample, 20,000 events were counted and statistical analysis was performed on three independent experiments while using the Mann and Whitney test (*p* < 0.05) (*N* = 3; *n* = 6).

### 4.7. Electric Impedance Measurement

Real-time electric impedance was analyzed over the whole cell culture duration by the XCelligence system (Roche, Basel, Switzerland) under a 5% CO_2_ atmosphere at 37 °C. The cells were seeded on a 96-wells-E-plate (Roche) with the bottoms surface area covered by interdigitated gold microelectrodes. The general principle is based on the presence of cells in the plate that affect the electric transmission between microelectrodes, and so leads to an increase of current resistance. This current resistance was called “cell index” or “impedance”, and it was directly dependent on cell line, culture media, and cell properties, such as cell number, cell proliferation, cell adherence intensity, or cell morphologic changes [34]. After cell seeding into replicates (ZR-75-1: 7500 cells/well), the E-plate was incubated in the XCelligence system that was set up in a CO_2_ incubator at 37 °C. The cells were allowed to grow for 24 h before treatment with K092A (1.22 µg/µL). Impedance was automatically analyzed every five minutes until 72 h and every hour until the end of the culture during the first 48 h of cell culture (including 24 h without treatment). 

### 4.8. Autophagy Measurement

Autophagy, which was directly correlated with the quantity of autophagosomes, was investigated at 4 h, 6 h, and 12 h post-treatment while using acridine orange assay and flow cytometry analysis. Acridine orange was used to stain autophagosome organelles (red fluorescence) and nucleic acids in cytoplasm and nucleus (green fluorescence). The percentage of autophagosomes (*R*; *R* = 100% for controls) was determined by normalization of the red fluorescence by the green fluorescence: *R* = FL3 INT/FL1 INT. The cells were seeded in replicates into 24-well plates (29,688 cells/well) and then treated. Cells were then stained with AO (5 µg/mL) for 10 min at 37 °C, collected (170 U trypsin/mL), washed, and analyzed by flow cytometry. For each sample, 20,000 events were counted and statistical analysis was performed on two independent experiments while using the Mann and Whitney test (*p* < 0.05) (*N* = 2; *n* = 4).

### 4.9. Immunocytochemistry Analysis

Tubulin and actin cytoskeletons were observed at 6 h and 48 h post-treatment while using a mouse anti-tyrosine tubulin antibody (Sigma: T9028; L’Isle d’Abeau Chesnes, France) and phalloidin staining (5 µg/mL), respectively. The cells were seeded in replicates on microscope cover glass slides into 24-well plates (ZR-75-1: 44,531 cells/well; MDA-Pca-2b: 29,688 cells/well) and treated. Cells were then fixed in 4% PFA for 10 min, washed in 1% BSA-PBS solution, and then incubated 1 h with rhodamine-phalloidin (1:200) or anti-tubulin (1:200). In the second case, the cells were then washed, incubated with secondary antibody (1:500; goat anti-mouse IgG Alexa fluor 488; Invitrogen: A11001; Thermo Fisher Scientific, Paris, France) for 1h in dark conditions, and then washed, while, after rhodamine-phalloidin incubation, the cells were only washed. Cover glass slides were finally mounted on microscope slides with a mounting solution containing DAPI (Prolong gold antifade with DAPI; Invitrogen P36935). Observations, pictures, and merge were taken with an Eclipse 80i microscope coupled to a DXM1200-C camera (Nikon, Champigny sur Marne, France). 

## 5. Conclusions

K092A and K092B isolated from male dogfish genital tract are potential antineoplastic marine peptides that are able to strongly inhibit the in vitro growth of ZR-75-1 and MDA Pca2b cells, respectively. The main as successive steps of K092A action in the ZR-75-1 cee-line are: (i) cytoskeleton disruption at 6 hpt; (ii) decrease of WST1, arrest in G0/G1, and decrease in the rate of S phase progression towards G2/M phases, increase in damaged membranes, and cell fragments at 48 hpt; and, (iii) increase of necrosis at 72 hpt. The main as successive steps of K092B action in MDA Pca2b cee-line are: (i) decrease of WST1, increase of necrosis and lysosomes/autophagosomes and of multinucleate cells at 6 hpt; (ii) arrest in G0/G1 and decrease in the rate of S phase progression towards G2/M phases; (iii) increase in damaged membranes and cell fragments at 48 hpt; and, (iv) prolonged necrosis at 72 hpt.

Both of the peptides appear to target a cytoskeleton element, quickly disrupt, and induce an early cytostatic fate, leading to a durable cell cycle perturbation and cell-number stabilization. Subsequently, the maintenance of cytoskeleton perturbation seems to be responsible for a later cytotoxic effect triggering non-apoptotic cell death mechanisms, such as necrosis and/or membrane disruption. Further investigations should be done in order to confirm this hypothesis. In any case, this study evidences that the dogfish could be a promising source of bioactive peptides against cancer cell growth.

## Figures and Tables

**Table 1:** Cell cycle repartition of ZR-75-1 cells in G0/G1, S, and G2/M (%).

Cycle	Sample	4 h	8 h	12 h	24 h	48 h	72 h
G0/G1	Control	60.7 ± 3.6	65.4 ± 3.3	70.9 ± 1.1	67.9 ± 2.1	74.3 ± 1.0	78.0 ± 1.2
	K092A	64.6 ± 6.8	65.5 ± 4.5	66.4 ± 2.9	71.5 ± 2.4	80.2 ± 1.0 *	66.6 ± 2.2 *
S	Control	13.7 ± 0.7	11.0 ± 1.1	8.0 ± 0.3	15.5 ± 1.5	14.0 ± 0.8	8.7 ± 2.4
	K092A	13.0 ± 2.7	10.0 ± 1.4	10.2 ± 2.4	11.5 ± 1.9 *	8.9 ± 0.9 *	14.9 ± 2.7 *
G2/M	Control	23.8 ± 2.8	22.1 ± 2.7	19.8 ± 1.2	16.6 ± 1.5	11.8 ± 1.2	9.0 ± 1.8
	K092A	21.0 ± 3.6	23.0 ± 3.0	22.3 ± 1.7	16.9 ± 1.6	8.7 ± 0.8 *	16.6 ± 2.5 *

Cell cycle repartition was measured by flow cytometry after propidium iodide (PI) staining at 4 h, 8 h, 12 h, 24 h, 48 h, and 72 hpt for three independent experiments on control cells and K092A-treated cells. Results in percentages were represented by mean ± SD and statistical analysis was performed using the Mann and Whitney test (* *p* < 0.05).

**Table 2:** Cell cycle repartition of MDA-Pca-2b cells in G0/G1, S, and G2/M (%).

Cycle	Sample	4 h	8 h	12 h	24 h	48 h	72 h
G0/G1	Control	55.4 ± 2.8	59.2 ± 1.3	49.8 ± 1.8	66.8 ± 0.6	72.5 ± 0.7	67.4 ± 0.8
	K092B	53.4 ± 4.2	64.5 ± 0.6 *	60.0 ± 2.1 *	60.5 ± 0.3 *	69.7 ± 1.1 *	73.2 ± 1.8
S	Control	14.7 ± 0.4	22.4 ± 0.4	22.9 ± 2.5	15.2 ± 0.4	10.5 ± 0.9	15.2 ± 0.3
	K092B	14.9 ± 0.0	14.3 ± 1.3 *	17.4 ± 2.2 *	16.7 ± 1.0	12.5 ± 2.8	10.2 ± 1.1 *
G2/M	Control	28.7 ± 1.3	20.8 ± 2.3	26.7 ± 3.7	16.8 ± 0.3	16.5 ± 1.2	17.7 ± 0.5
	K092B	30.9 ± 3.7	20.7 ± 1.4	19.0 ± 1.9 *	21.5 ± 0.7 *	17.1 ± 1.1	16.2 ± 3.3

Cell cycle repartition was measured by flow cytometry after propidium iodide (PI) staining at 4 h, 8 h, 12 h, 24 h, 48 h, and 72 hpt for three independent experiments on control cells and K092B-treated cells. Results in percentages were represented by mean ± SD and statistical analysis was performed while using the Mann and Whitney test (* *p* < 0.05).

**Table 3:** Apoptosis, necrosis, destabilized membranes, and cell fragments in ZR-75-1 control and K092A-treated cells at 24–72 h post-treatment (%).

Cell Death	Sample	24 h	48 h	72 h
Apoptosis	Control	1.5 ± 0.4	1.6 ± 0.3	1.5 ± 0.3
	K092A	1.4 ± 0.1	2.1 ± 1.0	3.7 ± 0.7 *
Necrosis	Control	3.8 ± 1.2	3.0 ± 1.3	3.3 ± 1.0
	K092A	4.1 ± 0.4	4.2 ± 1.2	10.8 ± 1.9 *
Destabilized membranes	Control	3.1 ± 1.1	5.4 ± 1.5	6.5 ± 1.3
	K092A	3.4 ± 1.7	8.4 ± 1.1 *	17.6 ± 3.3 *
Cell fragments	Control	2.2 ± 0.3	2.0 ± 1.1	3.5 ± 1.1
	K092A	2.7 ± 0.7	3.7 ± 0.1 *	8.7 ± 1.7 *

Data was obtained from three independent experiments (*N* = 3; *n* = 6), were represented by mean ± SD and statistical analyses were performed while using the Mann and Whitney test (* *p* < 0.05, treated vs. untreated control).

**Table 4:** Apoptosis, necrosis, destabilized membranes, and cell fragments in MDA-Pca-2b control and K092B-treated cells at 4–72 h post-treatment (%).

Cell Death	Sample	4 h	8 h	12 h	24 h	48 h	72 h
Apoptosis	Control	1.7 ± 0.4	2.2 ± 0.2	2.1 ± 0.2	2.0 ± 0.1	1.6 ± 0.5	2.0 ± 1.0
	K092B	2.2 ± 0.8	2.4 ± 0.4	3.0 ± 0.5	2.2 ± 0.7	2.3 ± 0.3	2.2 ± 0.7
Necrosis	Control	5.2 ± 0.4	2.8 ± 1.1	3.9 ± 0.9	2.7 ± 1.2	2.4 ± 0.1	3.5 ± 1.7
	K092B	5.8 ± 0.4	4.6 ± 0.5 *	12.7 ± 4.1 *	4.1 ± 0.6	3.8 ± 0.2	6.5 ± 0.8 *
Destabilized membranes	Control	7.8 ± 0.9	6.8 ± 0.2	7.0 ± 0.9	4.8 ± 1.1	3.5 ± 0.6	3.7 ± 0.2
	K092B	8.8 ± 0.3	6.2 ± 0.8	11.5 ± 1.8 *	6.6 ± 1.5	5.2 ± 1.1	7.6 ± 0.3 *
Cell fragments	Control	4.3 ± 0.2	4.5 ± 0.2	4.9 ± 0.4	3.1 ± 0.3	2.9 ± 0.1	3.4 ± 0.1
	K092B	4.5 ± 1.4	4.3 ± 2.1	4.7 ± 0.4	3.7 ± 0.4	5.7 ± 0.7 *	5.9 ± 0.7 *

Data was obtained from three independent experiments (*N* = 3; *n* = 6) represented by mean ± SD and statistical analyses were performed while using the Mann and Whitney test (* *p* < 0.05, treated vs. untreated control).

## Appendix A. In Vivo Inhibition of Cell-Derived Tumor in Nude Mice Model

In order to complete in vivo studies [33], the activity of K092B has been tested in Nude mice model using SK-OV-3 (ovarian cancer cell-line)-derived tumor. Briefly, 10 × 10^6^ cells/200 µL were subcutaneous injected. When tumors reached the size of 325 mm^3^, mice were randomized into a control group, receiving peptide buffer, and a treated group, receiving either a 25 mg/kg or a 130 mg/kg peptide intratumoral injection on every two days basis over an 8 days period. The tumor volume was registered and after 11 days, the mean tumor volume (MTV) and the ratio of the median tumor volume of treated group under the median tumor volume of control group (T/C) were determined. For the 25 mg/kg treated group, the results have shown the complete regression of the tumor for one mouse, a MTV of 1.69 ± 0.91 mm^3^ (*N* = 5) compared to 2.23 ± 0.25 mm^3^ for control (*N* = 5) and a T/C of 107.03%. For the 130 mg/kg treated group, the results have also shown the complete regression of the tumor for one mouse, a MTV of 1.52 ± 0.61 mm^3^ (*N* = 5) compared to 2.23 ± 0.25 mm^3^ for control (*N* = 5) and a T/C of 72.90%. Furthermore, frozen sections of tumors were analyzed by immunohistochemistry by using a rat IgG2a anti-mouse CD31 (PECAM-1: platelet endothelial cell adhesion molecule 1) (1:250; Cedarlane CL8930AP, clone 390) in order to follow angiogenesis of tumors. Results have shown an immunostaining at the center and at the periphery of tumors in the control group (*N* = 5) (Figure A1A) and, comparatively, a very low staining ay the center of tumors in the peptide-treated groups (*N* = 5 for each dose) (Figure A1B). At higher magnification, no cuboidal cells were stained in treated group (Figure A1D,E) compared to control group (Figure A1C) even if some isolated cells or trabecular tubules remained positive. These results suggested and antiangiogenesis activity of the K092B peptide.
